# Investigation of the Effectiveness of Surgical Treatment on Maxillary Medication-Related Osteonecrosis of the Jaw: A Literature Review

**DOI:** 10.3390/jcm10194480

**Published:** 2021-09-29

**Authors:** Kun-Jung Hsu, Szu-Yu Hsiao, Ping-Ho Chen, Han-Sheng Chen, Chun-Ming Chen

**Affiliations:** 1 School of Dentistry, College of Dental Medicine, Kaohsiung Medical University, Kaohsiung 80708, Taiwan; kjhsu1120@gmail.com (K.-J.H.); syhsiao2004@yahoo.com.tw (S.-Y.H.); phchen@kmu.edu.tw (P.-H.C.); 2 Department of Dentistry, Kaohsiung Medical University Hospital, Kaohsiung 80756, Taiwan; 3 Division of Dentistry for Child and Special Needs, Kaohsiung Medical University Hospital, Kaohsiung 80756, Taiwan; 4 Department of Dentistry, Kaohsiung Municipal Siaogang Hospital, Kaohsiung 80812, Taiwan; 5 Division of Oral and Maxillofacial Surgery, Department of Dentistry, Kaohsiung Medical University Hospital, Kaohsiung 80756, Taiwan

**Keywords:** MRONJ, BRONJ, maxilla, surgery, recurrence, literature review

## Abstract

Aim: Medication-related osteonecrosis of the jaw (MRONJ) occurs after exposure to medication (antiresorptive or antiangiogenic agents) for bone-related complications. It is more common in the mandible than in the maxilla. The present study investigated maxillary MRONJ in elderly patients through a meta-analysis. Methods: Keywords, including “MRONJ”, “maxilla”, and “surgery”, were entered into databases, including Embase, PubMed/MEDLINE, Cochrane Library, and ProQuest, which were searched systematically. Results: Investigating 77 studies, we found that 18 (2 case reports and 16 case series) papers conformed to the standards. The results revealed a 2.6:1 female-to-male ratio of disease occurrence. The average age of patients was 70.6 ± 5.5 years, and most patients were in the third stage (43.6%). The average time of medication usage was 50.0 ± 20.1 months. The pooled proportion of clinical efficacy of surgery was 86%. Conclusion: To prevent and manage MRONJ, all elderly patients should maintain proper oral hygiene and receive dental examinations regularly. Risk assessment and safety management of MRONJ should be performed by medical teams.

## 1. Introduction 

The World Health Organization states that most developed world countries characterize elderly people as aged 60 years and above. Most elderly people have at least one chronic disease and are at higher risk than others for developing osteoporosis [1,2]. Osteoporosis causes considerable bone loss in elderly people and bone fractures, due to the weakening of bones. The treatment of osteoporosis relies largely on bisphosphonates (BPs; mainly alendronate sodium (Fosamax^®^, Merck Sharp & Dohme Ltd., Kenilworth, NJ, USA)) and ibandronate (Boniva^®^, Roche Products Ltd., Basel, Switzerland) and denosumab, which inhibit osteoclast-mediated bone resorption to prevent bone fracture [3,4]. There are several functional differences between BPs and denosumab. BPs are micromolecules that remain at hydroxyapatite-binding sites on bone surfaces. When osteoclasts commence the resorption of BP-impregnated bones, BPs are released and bind with the farnesyl pyrophosphate synthase in osteoclasts, resulting in the apoptosis of osteoclasts. Denosumab is a human monoclonal antibody that works differently to BPs. It targets and binds with RANKLs, preventing activation of osteoclasts and the receptor activator of nuclear factor κB (RANK) on the surface of osteoclast precursors. The inhibition of RANKL–RANK interaction hinders osteoclast proliferation, functioning, and viability, thereby lowering bone resorption.

However, studies have found that intravenous or oral administration of BPs may cause bisphosphonate-related osteonecrosis of the jaw (BRONJ) [5,6]. The inhibition of normal osteoclasts is critical in the pathological mechanism for BRONJ—it reduces bone reformation, thus hindering the repair and adaptation of jaw bones. These medications may have an adverse effect on the oral mucosa and result in extensive damage the structures of the oral cavity, causing chewing and swallowing difficulties that lead to malnutrition and even increased risk of other chronic diseases. This can severely impair a patient’s oral function and quality of life.

Osteonecrosis of the jaw induced by drugs was first reported by Marx [5], who noted that 36 patients treated with pamidronate (Aredia^®^; Novartis Pharmaceuticals, East Hanover, NJ, USA) and zoledronate (Zometa^®^, Novartis Pharmaceuticals, East Haven, NJ, USA) experienced painful bone exposure in the mandible, maxilla, or both. An increasing number of studies have determined that osteonecrosis of the jaw is induced by other antiresorptive agents, including denosumab, a receptor activator of nuclear factor kappa-B ligand inhibitor; antiangiogenic agents, such as bevacizumab; and tyrosine kinase inhibitors, such as sunitinib and sorafenib [6,7,8,9,10,11]. Therefore, in 2014, the American Association of Oral and Maxillofacial Surgeons (AAOMS) proposed that osteonecrosis of the jaw related to medication be named medication-related osteonecrosis of the jaw (MRONJ) to differentiate it from BRONJ [12].

The ratio occurring MRONJ in the maxilla and mandible were between 1:2 and 1:3, possibly with the reason that mandible has the higher hardness and bone density. Most relevant research has focused on identifying the causes of MRONJ and determining the prognosis of its treatment; studies have typically focused on the mandible [13,14,15,16]. Only a few studies, such as case reports and case series, have discussed the results and prognosis of surgery for maxillary MRONJ. Accordingly, we inferred that the maxilla and mandible should be treated differently because their structures and the surrounding tissues differ. In old age, oral tissue aging, tooth wear, deteriorating periodontal disease, increased root caries, and reduced saliva secretion can cause numerous oral problems and increase the risk of chronic diseases, posing a major threat to health [17]. Therefore, we conducted a meta-analysis to investigate major clinical prognoses of surgical treatment for maxillary MRONJ in elderly patients.

## 2. Materials and Methods

### 2.1. Study Methods 

The study methods were based on the meta-analysis statistical evaluation and review guide of the Joanna Briggs Institute (JBI, 2014). In 2003, Marx [5] reported the first article about BRONJ, and then it raised many concerns for patients and doctors. Considering the disease process of BRONJ, we traced back to year 2000 for a literature review. From January 2000 to July 2021, we systematically searched English-language databases, including Embase, PubMed/MEDLINE, Cochrane Library, and ProQuest. Keywords including Medical Subject Headings and their synonyms such as “medication-related osteonecrosis of the maxilla” and “surgical treatment” were entered, and 77 papers were identified. Five papers were excluded due to duplication. Therefore, 72 papers were screened. 

### 2.2. Literature Search Strategies (Full Electronic Text)

The inclusion criteria for this study’s literature review were as follows:

(1) 2000–2021 English-language reference literature.
(2) Case report or case series.
(3) Patients with maxillary MRONJ or combined with mandibular MRONJ.
(4) An intervention of surgical treatment.
(5) Prognosis for the intervention outcome of wound healing, recurrence, or complication.
(6) Elderly people (age ≥ 60).

The exclusion criteria were as follows:

(1) Patients only with mandibular MRONJ.
(2) Osteonecrosis of the mandible unrelated to medication.
(3) Surgery not used as the major treatment but rather a conservative therapy, such as the use of antibacterial mouth rinse and full-body antibiotics.
(4) Animal experimentation.
(5) “Krokodil” drug or nucleoside reverse transcriptase inhibitor-related osteonecrosis.
(6) Reporting language other than English.

**Figure 1:**
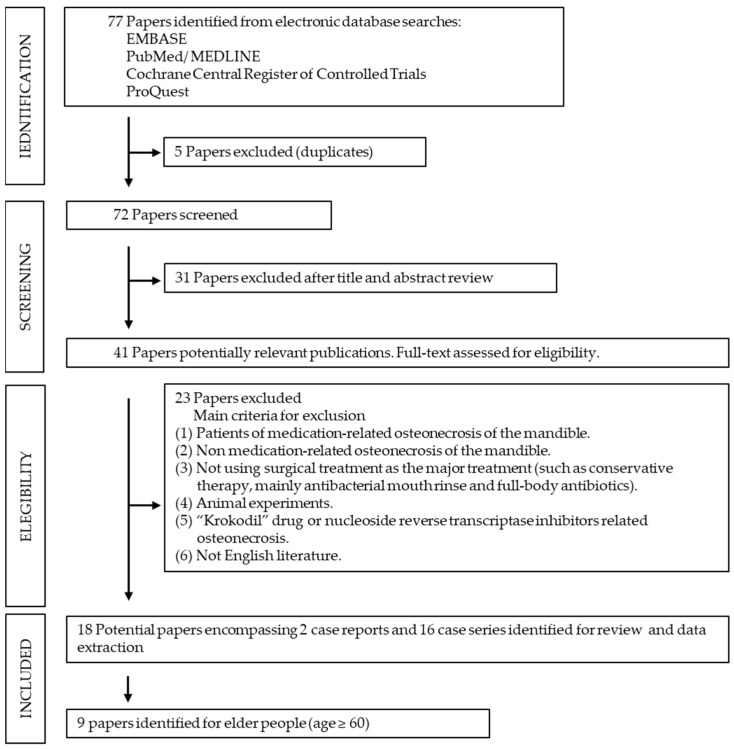
Process flow of study selection.

After excluding duplicate studies (5 papers) and those not conforming to the conditions (e.g., not written in English), we identified 72 papers. After title and abstract review, 31 references were excluded, leaving 41 potentially relevant publications. Their full text was assessed for eligibility, and 23 papers that did not conform to the main criteria were excluded. Finally, surgery was performed in the severe MRONJ in a total of 18 papers that were for the literature review [18,19,20,21,22,23,24,25,26,27,28,29,30,31,32,33,34,35] (Figure 1). The detailed data of elderly patients (age ≥ 60 years) were found in the 9 papers [20,21,22,23,25,26,27,28,29].

### 2.3. Literature Quality Assessment and Risk of Bias

Two independent reviewers screened the titles identified in the literature search, extracted the data, and analyzed the results. The use of a standard practice was proposed for extracting information regarding the studies, such as the author(s), year of publication, country, study design, and number of participants; patient data, such as gender (male/female) and mean age of patients; and disease data, such as osteonecrosis location, AAOMS stage, treatment type (surgery alone/surgery combined with adjuvant therapy), disease history, class of drugs (bisphosphonates/nonbisphosphonates), duration of drug exposure, follow-up time after treatment, and surgery outcomes of interest and effectiveness. One must be alert to the problem of bias in case series studies arising from the absence of a control group and the diversity of clinical and methodological approaches.

### 2.4. Data Extraction and Meta-Analysis

Two reviewers independently reviewed and extracted the original data from the 18 studies. After performing verification and reaching a consensus, MedCalc (version 19.6.4), a freely available Windows program, was used to conduct the meta-analysis and create the diagrams. Before combining the meta-analysis results, we conducted a heterogeneity test, which yielded an I2 between the studies. If I2 < 50%, then the different studies would have homogeneity and a fixed-effects model should be adopted. However, if the study designs of the combined studies had differences (such as different inclusion osteonecrosis locations, subjects, and intervention programs), then a random-effects model is recommended to avoid underestimating treatment variance. If I2 ≥ 50%, this would mean that the combined studies had heterogeneity and a random-effects model should be adopted. Sensitivity analysis can be further conducted, and unsuitable studies excluded before reanalysis. Furthermore, the use of subpopulation analysis is recommended to evaluate the effects of different subject populations. The estimation of combined effects considered the sample size of surgery efficacy and total sample of the studies. When different scales are used for the measurement indexes, the mean continuous variables and weighted sample number are adopted to estimate the combined effect. Furthermore, the fail-safe number test was used to determine publication bias. When the fail-safe number is larger than the tolerance level, this means that the possibility of publication bias is extremely low, and the results of the meta-analysis are more credible.

### 2.5. Eligibility Criteria

This article was written according to the PRISMA statement (Preferred Reporting Items for Systematic Reviews and Meta-Analyzes for Protocols).

### 2.6. Statistical Analysis

The major clinical prognoses were compared after patients with maxillary MRONJ received surgical treatment or surgical treatment combined with other treatments. The definition of successful treatment used in this study was as follows: (1) wound healing, (2) no recurrence, or (3) no complications. If one of these three outcomes was documented, then the surgical treatment was deemed effective. In the meta-analysis, the effect of clinical treatment was defined by the percentage of successful treatments and the 95% confidence interval (CI). The uncontrolled variables included in this study exhibited no significant differences. Accordingly, using the fixed-effects model was recommended. Other statistical analyses were performed using SPSS version 20 (SPSS Institute Inc., Chicago, DE, USA). 

### 2.7. Interpretation of Forest Plots

Forest plots are presented to summarize the data. Each horizontal line on a forest plot represents a case series included in the meta-analysis. The length of the line corresponds to a 95% CI of the corresponding case series’ effect estimate. The effect estimate is marked with a solid black square. The size of the square represents the weight of the corresponding study in the meta-analysis. The pooled estimate is marked with an unfilled diamond at the bottom of the forest plot. CIs of pooled estimates are displayed as a horizontal line through the diamond, where the line might be contained within the diamond if the CI is narrow. A statistically significant difference between both interventions studied was defined if their combined 95% CIs did not overlap. We considered a *p*-value of <0.05 to be statistically significant for the calculation of heterogeneity. Egger’s tests were used to assess the possibility of publication bias because they are useful adjuncts to meta-analysis; funnel plots were created therefrom. All proportional meta-analyses of case series studies were performed using MedCalc (version 19.6.4; MedCalc Software Ltd, Ostend, Belgium).

## 3. Results

### 3.1. Data Consolidation Analysis 

A total of 18 studies were included in this study, which comprised 2 case reports and 16 case series. Details of the case reports and case series are presented in the systematic literature review table. The kappa value of consistency between the two reviewers was 9.11 (*p* < 0.001). This indicates that the scores of the two reviewers had extremely high consistency and were significantly correlated.

Key extracted data of case studies are presented in Table 1. Demographic characteristics and clinical factors determined through consolidated analysis of patients with maxillary MRONJ are shown in Table 1. The design and major results of the 18 studies and the systematic literature review data are presented in Table 2. The date range used for the literature review was January 2000 to July 2021. The data were further analyzed in the meta-analysis. Some studies with incomplete data were excluded from the analysis. Data for the consolidated analysis were extracted from the studies where available (Table 1), and SPSS was used to estimate the means, standard deviations, and percentages. The results revealed that there were more female (65.6%) than male (25.2%) patients with maxillary MRONJ. The average age was 70.6 ± 5.5 years. The largest percentage of patients, 43.6%, were in AAOMS stage III [12]; this percentage was far higher than that of patients in stage II (34.4%), stage I (6.1%), or stage 0 (1.9%). Additionally, 42% of patients underwent surgical treatment, and 58% underwent a combination of surgical treatment and another conservative treatment. The most frequently occurring disease was osteoporosis (35.1%), followed by breast cancer (20.1%), multiple myeloma (15%), and prostate cancer (5.1%); the diseases of 18.2% of patients in the studies were unknown. Regarding medication, 79.1% of patients were taking bisphosphonates during the treatment period, with the most frequently administered being zoledronate (35.6%), followed by alendronate (10.2%), ibandronate (6.1%), risedronic acid (3.2%), and pamidronate (2.9%). Other treatment drugs were nonbisphosphonates, such as denosumab, which accounted for 11.4%. The average time of medication usage was 50.0 ± 20.1 months. The main prognosis outcomes were wound healing, recurrence, or complication.

### 3.2. Effectiveness of Surgery

**Figure 2:**
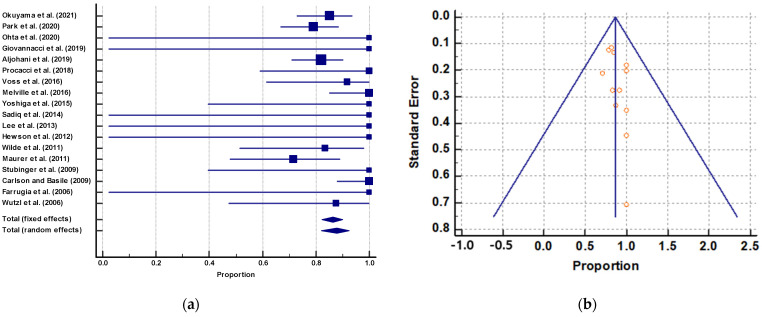
(**a**) A proportional meta-analysis of case report and case series studies regarding the clinical surgical efficacy in maxillary MRONJ. (**b**) Funnel plot of case series studies regarding the clinical efficacy in surgical therapy by Egger’s test.

The meta-analysis results for effectiveness of surgery are shown in Figure 2, with illustrations of case reports and case series published from 2006 to 2021. Proportional meta-analysis was performed on surgical outcomes (Figure 2). A significant difference between both interventions studied was defined as their combined 95% CI not overlapping. As demonstrated in the example, this analysis is an alternative approach to obtaining evidence of an intervention’s effects and plotting all available case reports and case series in the absence of clinical trials. For surgical treatment (18 papers), the pooled proportion of clinical efficacy (fixed-effects model) was 86% (95% CI, 82.24%–89.90%) for 2006–2021 studies with a total of 314 cases. The pooled proportion of clinical efficacy of surgery was 87.44% (95% CI, 79.89%–92.93%) for elderly people (nine papers) with a total of 111 cases.

The test of heterogeneity (I^2^ value = 37.34%, *p* = 0.0562) revealed no significant difference in the consistency (homogeneity) of clinical and methodological aspects between the studies included in the meta-analysis (Figure 2). Figure 2 presents a symmetric funnel plot based on data of the outcome of clinical efficacy in the case reports and case series determined through Egger’s test (*p* = 0.4141 > 0.05), which indicated no relationship between treatment effect and study size. These results suggest publication bias was not a factor in this meta-analysis.

## 4. Discussion

MRONJ is a serious adverse effect in some individuals taking medication for cancer or osteoporosis (drugs such as bisphosphonates (BPs), denosumab, and antiangiogenic agents), and it involves chronic osteonecrosis in the maxilla or mandible. In this meta-analysis, 79.1% of patients were taking BPs during the treatment period. Other treatment drugs were non-BPs, such as denosumab, which accounted for 11.4%. The average time of medication usage was 50.0 ± 20.1 months. Regarding prevalence of comorbidities, osteoporosis (31.5%) was predominant, followed by breast cancer (20.1%) and multiple myeloma (15%). The pathological mechanism of MRONJ is not entirely clear and can be multifactorial. The prevalence of MRONJ has exhibited an increase among elderly patients. As population aging becomes more pronounced, the oral hygiene and health of elderly people are crucial concerns. For consolidated analysis, data were extracted from the collected studies, and our results revealed that there were more elderly female patients with maxillary MRONJ. In both men and women, the average age was 70.6 years. Typical events that may precede MRONJ include acute periodontitis, denture misfit, wounds from invasive surgery, and other dentoalveolar surgeries. MRONJ is most frequently triggered by tooth extraction. In this meta-analysis, 8 of 18 articles presented that the trigger event of MRONJ was tooth extraction. Notably, approximately one-third of MRONJ cases are idiopathic without apparent cause. Such cases may possibly be attributable to subclinical traumas. Therefore, the risk factors of MRONJ include systemic factors (duration and dose of related drug, age, diabetes, and steroid) and local factors (thickness of mucosa, periodontal disease, periapical disease, and trauma of oral surgery) [7,12,22,25]. 

MRONJ is a potentially serious adverse event associated with high cumulative doses of antiresorptive or antiangiogenic drugs. A cessation of medication, such as the aforementioned drugs, is termed a “drug holiday” before invasive treatment. It is unclear whether a drug holiday before surgical treatments such as a tooth extraction is appropriate for BPs drug use. In an advisory statement, the American Dental Association in 2011 opined that a drug holiday is not required for patients receiving a relatively low cumulative dose of BPs (<2 years) or denosumab who may continue antiresorptive therapy during invasive dental treatment [36]. Wilde et al. reported no significant difference between treatment results, irrespective of whether or not treatment with BPs is continued [30]. Ottesen et al. [37] showed no evidence that supports the necessity of a drug holiday, even for patients on high-dose antiresorptive drugs because patient treatment outcomes vary widely. Hasegawa et al. [38] argued that no evidence suggests that a drug holiday before tooth extraction can lower MRONJ incidence. However, Korean scholars, such as Jung et al. [39], Kim et al. [40], and Kang et al. [41], have determined that drug holidays can effectively reduce MRONJ incidence. They recommend a drug holiday for 3–4 months before surgery. Melville et al. [25] adopted the drug holiday strategy in the treatment of an oroantral fistula. Di Fede et al. [42] proposed that no drug holiday is required for noncancer patients who use denosumab before or after surgery. However, patients taking BPs for over 3 years or less than 3 years and presenting with other systemic risk factors must have a drug holiday 1 week before and 4–6 weeks after surgery. Furthermore, patients with cancer require a drug holiday 1 week before and 4–6 weeks after surgery, and patients who use antiangiogenic drugs, such as bevacizumab, must have a drug holiday 6–7 weeks before and 4–6 weeks after surgery.

The denosumab does not accumulate in bone tissue and has a considerably shorter half-life than the bisphosphonates (28 days vs. 10–12 years) [43]. Hasegawa et al. [43] performed a multicenter retrospective study to investigate the effects (denosumab) of a short drug holiday (30 days) for tooth extraction in cancer patients. They found no significant difference in the occurrence of MRONJ between patients who had a drug holiday before tooth extraction and those who did not. Tooth extraction was significantly associated with development of MRONJ (odds ratio 4.69) in patients who had been taking oncologic doses of denosumab for a longer period. Therefore, it should be noted that effect of cumulative dose of the antiresorptive drug in the occurrence of MRONJ. The half-life of BPs in bones is over 10 years; therefore, short-term drug holidays may not be effective. Therefore, there is no clear recommendations can be made regarding a drug holiday with regard to the current state of research. 

Surgical treatments for maxillary MRONJ include sequestrectomy, debridement, resection, and immediate repair with/without flap reconstruction [42]. Nicolatou-Galitis et al. [44] suggested that a full-thickness mucoperiosteal flap should be used for the entire region of exposed bone in the resection of necrotic bone. In addition, disease-free edges should be exceeded. Healthy and bleeding bones should be visible for the resection of necrotic bone, and the sharp bone edges should be removed and smoothed. Suitable sutures with no tension must be used to achieve primary soft tissue closure for mucosa healing. Living bone and soft tissue should be retained as much as possible in surgery to promote wound healing, control jaw weakness, and maximize the probability of recovery [45]. Therefore, discriminating living from necrotic bone is critical. However, the boundary between necrotic and living bone is usually unclear, and the selection of surgical techniques depends on the experience and skill of the surgeon [45]. To improve surgical efficacy in this aspect, the latest method involves visualizing a fluorescent model [21,45] of living and necrotic bones (florescence-guided surgery). The florescent model displays the living and necrotic bones to make them more distinguishable, thus improving the surgical effectiveness. Aljohani et al. [22] reported a 93% healing rate after the first operation performed with fluorescence guidance, when compared with 79.6% for the cases treated without. However, Aljohani et al. [22] showed no significant difference between fluorescence guidance and wound healing.

Adjuvant therapies are currently recommended for maxillary MRONJ surgery. In a report by Park et al. [19], endoscopic sinus surgery was conducted with a combination of bone debridement for stage III patients. The MRONJ symptoms of approximately 84.2% of patients were improved, which was significantly higher than that of dentoalveolar surgery alone (37.5%). Aljohani et al. [22] recommended the removal of necrotic bone followed by closure with mucoperiosteal flap was reliable for MRONJ treatment. The buccal fat pad (BFP) flap is effective for the closure of MRONJ-related oroantral communications. Complete mucosal healing occurred in 85.7% of patients when the BFP flap was added for the formation of a double-layer closure. The usage of platelet-rich plasma (PRP) to improve the healing of postoperative wounds is gaining popularity. Melville et al. [25] applied the BFP flap and radical sinusotomy approaches with the administration of PRP as a growth factor. Alternatively, PRP can be used to facilitate the regeneration of defective bone and neighboring soft tissue after tooth extraction, which reduces the risk of MRONJ. After placement above the BFP flap, primary closure can be conducted. Melville et al. [25] reported that their patients exhibit 100% resolution of MRONJ and sinusitis. In this meta-analysis, the use of BFP flaps to close maxillary defects with two layers is highly recommended to achieve the surgery efficacy [25]. 

Moreover, several methods may be employed as alternative treatment options for enhancing MRONJ bone healing. Low-level laser therapy is a promising supplementary treatment for MRONJ because it facilitates biological effects, such as inflammation and angiogenesis, and simultaneously increases the inorganic substrate and facilitates wound healing [46]. The recombinant human bone morphogenic protein-2 (rhBMP-2) can induce osteogenesis and is extensively used for bone defect treatment [47]. After sequestrectomy, the carrier (absorbable collagen sponge) containing rhBMPs is placed in the defect area. Park et al. reported that the adjusted odds ratios indicates a better healing tendency of platelet-rich fibrin (L-PRF) and recombinant human bone morphogenic protein-2 (rhBMP-2) insertion relative to sequestrectomy alone during all the follow-up periods; however, this did not reach significance [48]. Hyperbaric oxygen (HBO) is a well-established treatment to promote angiogenesis and wound healing [48]. However, Farrugia et al. reported that the benefits of HBO as a supplementary method to improve MRONJ healing in conventional treatment remain unclear [34]. In this meta-analysis, there is a lack of measurable effects of HBO therapy in MRONJ. Therefore, it requires further investigation to be an adjuvant treatment for MRONJ. 

Periosteal reaction is a nonspecific radiographic finding that indicates periosteal irritation. To clarify the clinical significance of the periosteal reaction in wound healing, Kojima et al. [49] investigated the relationship between periosteal reaction and treatment outcome of MRONJ. They found the cure rate after surgical treatment decreased in cases with periosteal reaction. The periosteal reaction represented a more destructive lesion formed because of obstacles to bone remodeling, associated with the apoptosis of osteoclasts. However, Okuyama, et al. [18] reported that periosteal reaction was of no significance for the prognosis of maxillary MRONJ. Hence, the significance of periosteal reaction in wound healing for MRONJ has been controversial.

It is desirable to analyze existing data so that physicians and health professionals may have a reference for the current state of knowledge. For this reason, conducting a proportional meta-analysis of case series studies with a comprehensive systematic search of uncontrolled studies (i.e., case series) is suggested when there are no clinical trials in the literature that answer questions about clinical interventions. However, investigators and policymakers should be extremely cautious in interpreting such results because many flaws may be evident in the internal validity of this type of study, specifically, bias in case series studies arising from the absence of a control group and the diversity of clinical and methodological approaches. Nevertheless, the evidence provided from a proportional meta-analysis of case series studies should only be used until appropriate clinical trials are conducted. Okuyama et al. [18] reported the complete wound healing rate of maxillary MRONJ is 85.2%. Our analysis also shows that the effectiveness of surgery is up to 86%. Furthermore, we identified clinical outcomes homogeneity in case series studies. The funnel plot of the case series suggests the possibility that publication bias may not occur given the symmetry; however, the clinical efficacy outcome exhibited relatively little heterogeneity. This suggests that the case series were far more consistent in patient selection and treatment protocol. 

In this study, we used a proportional meta-analysis of case studies, specifically case report or case series studies of maxillary MRONJ, in order to evaluate the effectiveness of surgery as treatment. Our evidentiary level was relatively low, which affected the ability to determine the efficacy and safety of interventions, surgical procedures, and prevention programs. Nonetheless, this alternative analysis can help surgeons, physicians, and health professionals to make provisional decisions in conjunction with their clinical expertise and the patient’s wishes and circumstances. We recommend that healthcare professionals weigh the benefits and risks of surgery under this approach and take account of the patient’s values and preferences. Moreover, we recommend further research involving high-quality primary studies and ethical and methodologically sound clinical trials.

## 5. Conclusions

In this study, the average age of patients was 70.6 years and with a 2.6:1 female-to-male ratio. Most patients were in the third stage (43.6%). Clinical efficacy of surgery was up to 86%. In order for the MRONJ to be prevented and managed, all elderly patients were suggested to maintain a proper oral hygiene routine and receive routine dental examination. In this study, tooth extraction is considered to be a trigger event. Therefore, a detailed inquiry with risk factors for MRONJ is critical before tooth extraction or other dentoalveolar surgery. Patients’ medical histories (osteoporosis or malignancy) and past medication usage (BPs or non-BPs) should be fully understood, and they should be evaluated by a medical team (including a surgical option).

## Figures and Tables

**Table 1:** Demographic characteristics and clinical factors from consolidated analysis of patients with maxillary MRONJ (*n* = 314).

	Maxillary MRONJ (*n* = 314)	
	*n*	(%) ^a^
Gender		
Males	79	(25.2%)
Females	206	(65.6%)
NA	29	(9.2%)
Age (years), mean ± SD	70.6 ± 5.5	
Osteonecrosis location		
Maxilla	313	(99.7%)
Maxilla + mandible	1	(0.3%)
Stage ^b^		
0	6	(1.9%)
I	19	(6.1%)
II	108	(34.4%)
III	137	(43.6%)
NA	44	(14%)
Treatment		
Surgery alone	132	(42.0%)
Surgery combined adjuvant therapy	182	(58.0%)
Disease history		
Osteoporosis	110	(35.1%)
Breast cancer	63	(20.1%)
Multiple myeloma	47	(15.0%)
Prostate cancer	16	(5.1%)
Lung cancer	2	(0.6%)
Renal cancer	2	(0.6%)
Cervical cancer	1	(0.3%)
Thyroid cancer	1	(0.3%)
Rectal cancer	1	(0.3%)
Leukemia	1	(0.3%)
Other malignancies	13	(4.1%)
NA	57	(18.2%)
Drugs		
Bisphosphonates		
Zoledronate	112	(35.6%)
Alendronate	32	(10.2%)
Ibandronate	19	(6.1%)
Risedronate	10	(3.2%)
Pamidronate	9	(2.9%)
Zoledronate combined other drugs	9	(2.9%)
NA	57	(18.2%)
Non bisphosphonates		
Denosumab or denosumab combined with other drugs	36	(11.4%)
NA	30	(9.5%)
Duration of drug exposure (months), mean ± SD	50.0 ± 20.1	
Follow up time of post treatment (months), mean ± SD	8.4 ± 6.6	
Treatment effectiveness		
Wound complete healing	270	(86%)
Impaired wound healing or recurrence	44	(14%)

NA: not available; MRONJ: medication-related osteonecrosis of the jaw; SD: standard deviation. ^a^ May not total 100% due to rounding. ^b^ The severity of clinical stage was defined by American Association of Oral and Maxillofacial Surgeons (AAOMS) stage (2014).

**Table 2:** Surgical treatment of patients with maxillary MRONJ: a systematic review of case report and case series.

Author [Ref]/Country (Year)	Study Design (*n*)	M (N)/F (N) Age (Years): Mean ± SD (Min.-Max.)	Therapy	Disease (*n*)	Drugs (*n*)	Treatment Outcome
Okuyama et al. [18]/Japan (2021)	Multiple center Case series (*n* = 54)	M (17)/F (37) Age: 73 (48–89)	Resection alone (*n* = 54)	Osteoporosis (*n* = 28) Cancer (*n* = 26)	Bisphosphonates (*n* = 34) Denosumab (*n* = 20)	No recurrence (*n*= 46) Residual necrotic bone (*n* = 8)
Park et al. [19]/Korea (2020)	Case series (*n* = 62)	M (10)/F (52) Age: 72.1 ± 11.3 (43–92)	Resection alone (*n* = 9) Resection + PRF (*n* = 19) Resection + PRF + BMP (*n* = 34)	Osteoporosis (*n* = 46) Breast cancer (*n* = 10) Multiple myeloma (*n* = 5) Cervical cancer (*n* = 1)	Alendronate (*n* = 27) Risedronate (*n* = 8) Pamidronate (*n* = 4) Ibandronate (*n* = 11) Zolendronate (*n* = 9) Others (*n* = 3)	Resection: Resolution (*n* = 5)/ No resolution (*n* = 4) Resection + PRF: Resolution (*n* = 14)/ No resolution (*n* = 5) Resection + PRF + BMP: Resolution (*n* = 30)/ No resolution (*n* = 4)
Ohta et al. [20]/Japan (2020)	Case report (*n* = 1)	M (1) Age: 79	Resection alone (*n* = 1)	Bone metastatic prostate cancer	Zolendronate + denosumab	No recurrence/ No complications
Giovannacci et al. [21]/Italy (2019)	Case report (*n* = 1)	F (1) Age: 80	Resection + auto-fluorescence + LLLT	Breast cancer	Denosumab	No recurrence No complications
Aljohani et al. [22]/Germany (2019)	Case series (*n* = 72)	M (26)/F (46) Age: 72 ± 9.6	Resection (*n* = 32) Resection + BPF (*n* = 14) Resection + fluorescence guided (*n* = 5)	Breast cancer (*n* = 28) Multiple myeloma (*n* = 15) Prostate cancer (*n* = 10) Osteoporosis (*n* = 6) Others (*n* = 13)	Zoledronate (*n* = 45) Pamidronate (*n* = 1) Ibandronate (*n* = 3) Combination of bisphosphonates (*n* = 10) Denosumab (*n* = 4) Zoledronate and denosumab (*n* = 7) Denosumab and ibandronate (*n* = 1) Denosumab, zoledronate, pamidronate (*n* = 1)	No recurrence in 82.2% (65/79) of the lesions
Procacci et al. [23]/Italy (2018)	Case series (*n* = 7)	M (1)/F (6) Age: 66 ± 10.6 (51–79)	Resection + BPF (*n* = 7)	Breast cancer (*n* = 2) Osteoporosis + rheumatoid arthritis (*n* = 2) Leukaemia (*n* = 1) Severe osteoporosis (*n* = 2)	Zoledronate (*n* = 3) Risedronate (*n* = 2) Denosumab (*n* = 1) Risedronate (*n* = 1)	No recurrence No complications
Voss et al. [24]/Germany (2016)	Case series study (*n* = 12)	M (2)/F (10) Age: 67.0 (55–62)	Resection (*n* = 12)	Breast cancer (*n* = 5) Multiple myeloma (*n* = 5) Lung cancer (*n* = 1) Osteoporosis (*n* = 1)	Bisphosphonate: Zoledronate (*n* = 5) Pamidronate (*n* = 3) Ibandronate (*n* = 2) Alendronate (*n* = 1) Clodronate (*n* = 1)	No recurrence (*n* = 11) Recurrence (*n* = 1)
Melville et al. [25]/USA (2016)	Case series (*n* = 23)	M (3)/F(20) Age = 68.3 ± 12.3	Resection + BFP (*n* = 5) Resection + BFP + PRP (*n* = 18)	Osteoporosis (*n* = 23)	Zoledronate (*n* = 19) Zoledronate and avastin (*n* = 1) Alendronate and ibandronate (*n* = 1) Alendronate (*n* = 1) Denosumab (*n* = 1)	No recurrence (*n* = 23)
Yoshiga et al. [26]/Japan 2015	Case series (*n* = 4)	F (4) Age: 69.8 ± 11.2 (58–85)	Resection (*n* = 4)	Osteoporosis (*n* = 1); Breast cancer (*n* = 3)	Bisphosphonate (oral) (*n* = 1)/120 Bisphosphonate (IV) (*n* = 3)/	No recurrence (*n* = 4)
Sadiq et al. [27]/UK (2014)	Case series (*n* = 1)	F (1) Age = 83	Resection (*n* = 1)	Osteoporosis	Alendronate Bendroflumethiazide Enalapril	No recurrence (*n* = 1)
Lee et al. [28]/Taiwan (2013)	Case series (*n* = 1)	F (1) Age = 76	Resection (*n* = 1)	Osteoporosis	Zoledronate	No recurrence (*n* = 1)
Hewson et al. [29]/Australia (2012)	Case series (*n* = 1)	F (1) Age = 64	Resection (*n* = 1)	Multiple myeloma	Pamidronate + zoledronate	No recurrence (*n* = 1)
Wilde et al. [30]/Germany (2011)	Case series (*n* = 12)	M (4)/F (8) Age = (48–93)	Resection +BFP	Multiple myeloma (*n* = 3)Breast cancer (*n* = 5) Prostate cancer (*n* = 3); Thyroid cancer (*n* = 1)	Zolendronate (*n* = 6) Zolendronate + bondronate (*n* = 2) Zolendronate + pamindronate (*n* = 2) Zolendronate + pamindronate + bondronate (*n* = 2)	No recurrence (*n* = 10) Recurrence (*n* = 2)
Maurer et al. [31]/Germany (2011)	Case series (*n* = 21)	M (5)/F (16) Age: 69.0 ± 10.2 (48–91)	Resection	Breast cancer (*n* = 6) Multiple myeloma (*n* = 8) Osteoporosis (*n* = 3) Lung cancer (*n* = 1) Prostate cancer (*n* = 1) Rectal cancer (*n* = 1) Renal cancer (*n* = 1)	Alendronate (*n* = 3)/ibandronate (*n* = 3)/zoledronate (*n* = 15)/	No recurrence (*n* = 15) Recurrence (*n* = 6)
Stubinger et al. [32]/Slovenia (2009)	Case series (*n* = 4)	M (3)/F (1) Age: 70.0 ± 9.6 (56–77)	Resection + Er:YAG laser ablation	Prostate cancer (*n* = 1) Myeloma (*n* = 2) Renal cell carcinoma (*n* = 1)	Zoledronate (*n* = 4)	No recurrence (*n* = 4)
Carlson and Basile [33]/USA (2009)	Case series (*n* = 29)	NA	Resection (*n* = 29)	Breast cancer Multiple myeloma Prostate cancer Osteoporosis Other malignancies	Alendronate Risedronate Zoledronate Pamidronate	No recurrence (*n* = 29)
Farrugia et al. [34]/USA (2006)	Case series (*n* = 10)	NA	Resection	Breast cancer (*n* = 3) Multiple myeloma (*n* = 5) Paget’s disease (*n* = 1) Osteoporosis (*n* = 1)	Pamidronate + zoledronate	No recurrence (*n* = 1)
Wutzl et al. [35]/Austria (2006)	Case series (*n* = 8)	M (5)/F (3) Age: 66 ± 9 (51–76)	Resection (*n* = 7) Refused surgery (*n* = 1)	Breast cancer (*n* = 3) Multiple myeloma (*n* = 5)	Zoledronate (*n* = 5) Pamidronate (*n* = 1) Pamidronate/zoledronate (*n* = 2)	No recurrence (*n* = 7) Recurrence (*n* = 1)

NA: not available. Abbreviations: M, male; F, female; Min., minimum; Max., maximum; PRF, leukocyte-rich and platelet-rich fibrin; BMP, bone morphogenetic protein; AF, surgical resection auto-fluorescence guided; LLLT, low-level laser therapy; BFP, buccal fat pad; PRP, platelet-rich plasma.

## Data Availability

The data used to support the findings of this study are included within the article. The data used to support the findings of this study are available from the corresponding author upon request.
